# Optimizing Vagus Nerve Stimulation Parameters in Pediatric Drug-Resistant Epilepsy: A Retrospective Two-Center Study

**DOI:** 10.3390/children12091222

**Published:** 2025-09-12

**Authors:** Müge Baykan, Özge Baykan Çopuroğlu, Elif Didinmez Taşkırdı, Pınar Gençpınar, Nihal Olgaç Dündar

**Affiliations:** 1 Department of Pediatric Neurology, Rize Training and Research Hospital, Recep Tayyip Erdoğan University, Rize 53020, Turkey; 2 Department of Therapy and Rehabilitation, Incesu Ayşe and Saffet Arslan Vocational School of Health Services, Kayseri University, Kayseri 38560, Turkey; ozgebaykancopuroglu@kayseri.edu.tr; 3 Department of Pediatric Neurology, Izmir City Hospital, Izmir 35530, Turkey; edidinmez@hotmail.com; 4 Department of Pediatric Neurology, Faculty of Medicine, Izmir Katip Çelebi University, Izmir 35620, Turkey; pinargencpinar@gmail.com (P.G.); nodundar@gmail.com (N.O.D.); 5 Neuroscience Research Center, Faculty of Medicine, Izmir Katip Çelebi University, Izmir 35620, Turkey

**Keywords:** pediatric epilepsy, drug-resistant epilepsy, vagus nerve stimulation, stimulation parameters, seizure control

## Abstract

**Highlights:**

**What are the main findings?**

- In a pediatric cohort with drug-resistant epilepsy, 76.9% of patients achieved ≥50% seizure reduction following vagus nerve stimulation (VNS).
- Optimal clinical response was observed at an output current of ~1.5 mA and a duty cycle of 10%, beyond which additional increases did not improve outcomes.

**What is the implication of the main finding?**

- Children may respond to lower-intensity VNS settings than adults, suggesting a need for age-specific programming protocols.
- Early stabilization at effective thresholds may reduce unnecessary stimulation and improve treatment tolerability in pediatric epilepsy care.

**Abstract:**

Objectives: Drug-resistant epilepsy (DRE) remains a major challenge in pediatric neurology, as many children fail to achieve seizure control despite appropriate medications. Vagus nerve stimulation (VNS) offers an effective adjunctive treatment; however, optimal stimulation parameters for children are not well defined and are often extrapolated from adult protocols. This retrospective two-center cohort study aimed to evaluate the clinical effectiveness of VNS in pediatric DRE and to determine stimulation thresholds—particularly output current and duty cycle—most strongly associated with seizure reduction. Methods: Fifty-two pediatric patients (aged 0–18 years) with DRE who underwent VNS implantation and were followed for at least 12 months were retrospectively analyzed. Stimulation frequency and pulse width were fixed at 30 Hz and 250 µs, while output current and duty cycle were titrated based on clinical response. Seizure outcomes were derived from caregiver-maintained seizure diaries and confirmed during structured follow-up visits. Treatment response was defined as a ≥50% reduction in seizure frequency compared to baseline. Results: At 12 months post-implantation, 76.9% of patients achieved ≥ 50% seizure reduction, 32.7% experienced ≥ 90% reduction, and 11.5% attained complete seizure freedom. Optimal outcomes were associated with output currents of approximately 1.5 mA and duty cycles of 10%. Conclusions: VNS is a highly effective and well-tolerated treatment for pediatric DRE. Stabilization at an output current of 1.5 mA and a 10% duty cycle may serve as a clinically useful programming target. These findings support the use of individualized, age-specific stimulation strategies to optimize outcomes in pediatric VNS therapy.

## 1. Introduction

Epilepsy is one of the most common chronic neurological disorders in childhood, affecting nearly 0.5–1% of the pediatric population globally [1]. Despite significant advances in diagnostic capabilities and pharmacological therapies, approximately 20–30% of children with epilepsy continue to experience seizures that are refractory to antiseizure medications (ASMs) [2]. This condition, defined as drug-resistant epilepsy (DRE) by the International League Against Epilepsy (ILAE), poses a substantial burden to patients, families, and healthcare systems [3]. Beyond the increased risk of physical injury and sudden unexpected death in epilepsy (SUDEP), children with uncontrolled seizures often face neurocognitive delay, behavioral disorders, reduced quality of life, and stigmatization, which together impair long-term developmental outcomes [4,5].

For patients who are not candidates for resective surgery or fail to respond to it, neuromodulation techniques such as vagus nerve stimulation (VNS) have emerged as viable adjunctive treatment options. Since its initial FDA approval in 1994, VNS has become an established therapy for focal and generalized epilepsies that are pharmacoresistant [6]. Numerous studies have demonstrated that VNS can significantly reduce seizure frequency, improve alertness, stabilize mood, and promote social interaction in both adults and children [7,8]. The therapeutic effect of VNS is believed to stem from afferent vagal activation of the nucleus tractus solitarius, followed by widespread modulation of cortical and subcortical networks including the thalamus, limbic system, and locus coeruleus [9,10].

Although the basic mechanism and clinical utility of VNS are well documented, optimal stimulation parameters remain a subject of ongoing investigation, especially in pediatric populations. Standard VNS programming involves adjustment of four key variables: output current, duty cycle, frequency, and pulse width. However, most existing guidelines and titration protocols are based on adult studies or manufacturer recommendations, with limited pediatric-specific evidence [11,12]. This is a notable limitation, as children differ from adults in several key respects, including neurodevelopmental status, seizure semiology, autonomic tone, and sensitivity to neuromodulation [13].

Recent findings suggest that children may achieve seizure control at lower stimulation thresholds than adults, potentially due to greater neural plasticity or differences in vagal nerve fiber composition and excitability [14,15]. Conversely, overstimulation in pediatric patients may lead to adverse effects such as dysphonia, dysphagia, or sleep disturbances, which can undermine adherence and therapeutic success [16]. These concerns underscore the need for more precise, individualized programming strategies that consider developmental and physiological differences.

Among all modifiable parameters, output current and duty cycle are the two most clinically adjustable and impactful in shaping VNS response. While frequency and pulse width are generally standardized across institutions (commonly 30 Hz and 250 μs), output current and duty cycle are typically titrated over time based on clinical response and tolerability. However, there remains no clear consensus regarding the minimum effective dosing or the optimal timing and trajectory of stimulation escalation in pediatric patients [17]. Furthermore, few studies have systematically analyzed how these parameters relate to seizure reduction outcomes using robust statistical modeling in real-world pediatric cohorts.

In light of these knowledge gaps, the present study was designed as a retrospective observational analysis to evaluate the clinical associations between stimulation parameters and seizure outcomes in a cohort of pediatric patients with drug-resistant epilepsy across two tertiary epilepsy centers. By holding stimulation frequency and pulse width constant, this analysis specifically explored output current and duty cycle levels most strongly associated with favorable seizure reduction. The goal was to identify clinically relevant stimulation ranges that may inform pediatric-specific VNS programming protocols.

## 2. Materials and Methods

### 2.1. Study Design and Ethical Considerations

This retrospective observational cohort study was conducted at two tertiary epilepsy centers in Türkiye, both of which maintain specialized pediatric neurology clinics and standardized VNS protocols. The study aimed to evaluate the association between stimulation parameters and clinical response in pediatric patients with DRE. Ethical approval was obtained from the institutional review boards of both centers. The research adhered to the principles of the Declaration of Helsinki and national regulations for retrospective data analysis. All patient data were anonymized prior to analysis to ensure confidentiality and compliance with data protection standards.

### 2.2. Patient Selection

The study population included pediatric patients aged 0 to 18 years who met the ILAE criteria for DRE, defined as the failure of at least two appropriately chosen and adequately dosed antiseizure medications [3]. Inclusion required VNS implantation between January 2012 and December 2022 and a minimum follow-up period of 12 months post-implantation. Patients were excluded if they had undergone concurrent resective epilepsy surgery during the study period, experienced changes in stimulation frequency or pulse width during follow-up or had incomplete clinical records. Baseline seizure frequency and epilepsy types were extracted and reported (see Table 1).

### 2.3. VNS Programming and Stimulation Parameters

All patients received commercially available VNS systems, with initial stimulation initiated within two weeks following device implantation. In alignment with standard pediatric practice, stimulation frequency and pulse width were fixed at 30 Hz and 250 microseconds (µs), respectively, for all participants.

Output current was initiated at 0.25 mA and increased in increments of 0.25 mA every 2–4 weeks, depending on clinical response and tolerability. The duty cycle was started at 10% and adjusted gradually (typically in steps of 5–10%) if seizure control was insufficient. Device settings were checked at each follow-up visit, and titration decisions were made collaboratively by the treating physician and caregivers, balancing seizure response and tolerability. Adverse events were not systematically recorded; however, clinical notes occasionally documented transient effects such as hoarseness or mild swallowing difficulties.

### 2.4. Data Collection and Outcome Measures

Demographic and clinical information—including age at VNS implantation, epilepsy duration, seizure type, and baseline seizure frequency—was extracted from electronic medical records and operative reports. Stimulation data were retrieved from programming logs. The primary outcome measure was clinical response, defined as a ≥50% reduction in seizure frequency compared to baseline, assessed at the 12-month follow-up.

Seizure outcomes were primarily derived from caregiver-maintained seizure diaries. To improve reliability, these diaries were verified and cross-checked during structured clinical interviews at each follow-up visit. Only patients with consistent and complete documentation were included in the final analysis.

### 2.5. Statistical Analysis

Descriptive statistics were calculated for all demographic and clinical variables. Continuous data were presented as means ± standard deviations, and categorical variables as frequencies and percentages.

The association between stimulation parameters and clinical response was analyzed using a generalized linear mixed model (GLMM) with a logit link function. Clinical response (responder vs. non-responder) was treated as the binary dependent variable. Independent variables included output current (mA), duty cycle (%), age at implantation, and epilepsy duration. A random intercept for center was included to account for clustering effects. The GLMM approach was chosen due to its robustness for hierarchical data and moderate sample sizes.

Model performance was assessed using −2 log-likelihood values, Akaike Information Criterion (AIC), and Bayesian Information Criterion (BIC). Discrimination was evaluated with receiver operating characteristic (ROC) analysis, and calibration was assessed using the Hosmer–Lemeshow test. Odds ratios (ORs) with 95% confidence intervals (CIs) were reported. Statistical significance was defined as *p* < 0.05.

Analyses were performed using IBM SPSS Statistics for Windows, version 25.0 (IBM Corp., Armonk, NY, USA).

## 3. Results

### 3.1. Patient Characteristics

A total of 52 pediatric patients with DRE were included in the final analysis. The mean age at VNS implantation was 9.2 ± 4.5 years, and the average duration of epilepsy prior to surgery was 5.8 ± 3.1 years. The cohort comprised 57.7% males (n = 30) and 42.3% females (n = 22). Regarding epilepsy etiology, 32.7% of cases were classified as genetic, 21.2% structural, 5.8% infectious, and 40.3% of unknown or other origin. Generalized seizure types were more prevalent (53.9%) than focal seizures (46.1%). The mean baseline seizure frequency was 54.7 ± 21.3 seizures per month, with generalized seizures tending to occur more frequently than focal events. Baseline clinical and demographic characteristics are summarized in Table 1.

### 3.2. Stimulation Parameters and Programming Progression

**Figure 1:**
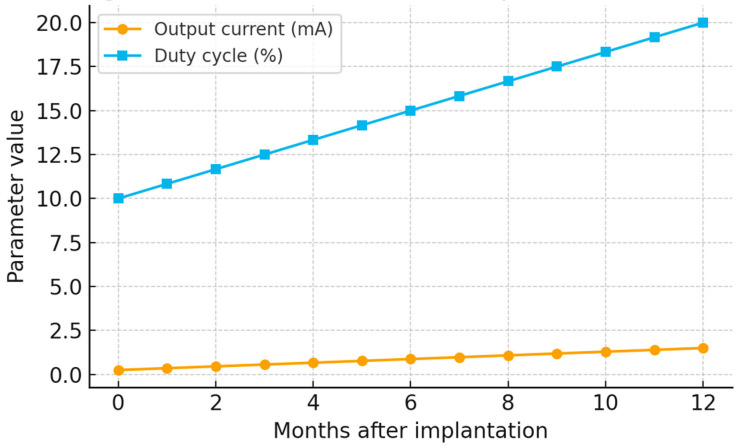
Line graph showing progressive increases in output current and duty cycle over the 12-month follow-up period.

All patients were initiated on standardized VNS settings with a fixed stimulation frequency of 30 Hz and a pulse width of 250 μs. The initial output current and duty cycle were uniformly set at 0.25 mA and 10%, respectively. These parameters were titrated over time based on individual clinical response and tolerability. By 12 months, output current was increased to an average of 1.5 mA (range: 0.75–2.25 mA), while duty cycle rose to a mean of 20% (range: 10–30%). The evolution of stimulation parameters is detailed in Table 2 and visualized in Figure 1, which have been revised for internal consistency.

### 3.3. Clinical Outcomes

**Figure 2:**
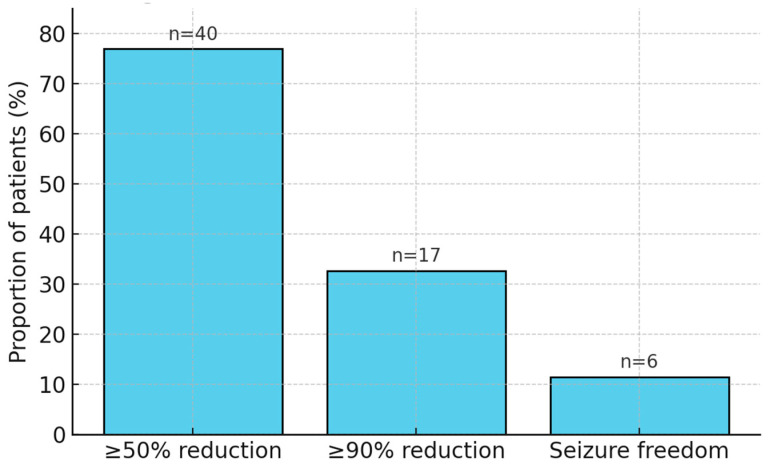
Bar graph illustrating response categories (≥50%, ≥90%, seizure freedom) at 12 months.

At the 12-month follow-up, 76.9% of patients (n = 40) achieved a ≥50% reduction in seizure frequency. Among these responders, 32.7% (n = 17) attained a ≥90% reduction, and 11.5% (n = 6) became seizure-free. Clinical responders were most consistently clustered around an output current of ~1.5 mA and a duty cycle of ~10%, with diminishing returns observed at higher levels. These findings highlight the substantial efficacy of VNS therapy in the pediatric DRE population. Outcome distribution is presented in Table 3 and illustrated in Figure 2.

### 3.4. Predictors of Clinical Response

To identify predictors of clinical success, a GLMM was applied. Output current and duty cycle were both statistically significant predictors of response. Each 1 mA increase in output current increased the odds of a ≥50% reduction in seizure frequency by 4.48-fold (OR: 4.48; 95% CI: 2.29–8.76; *p* < 0.001). Each 1% increase in duty cycle raised the odds by 11% (OR: 1.11; 95% CI: 1.04–1.27; *p* = 0.002). Neither age at implantation nor epilepsy duration demonstrated significant associations. Full model details are shown in Table 4.

### 3.5. Model Performance and Discrimination

**Figure 3:**
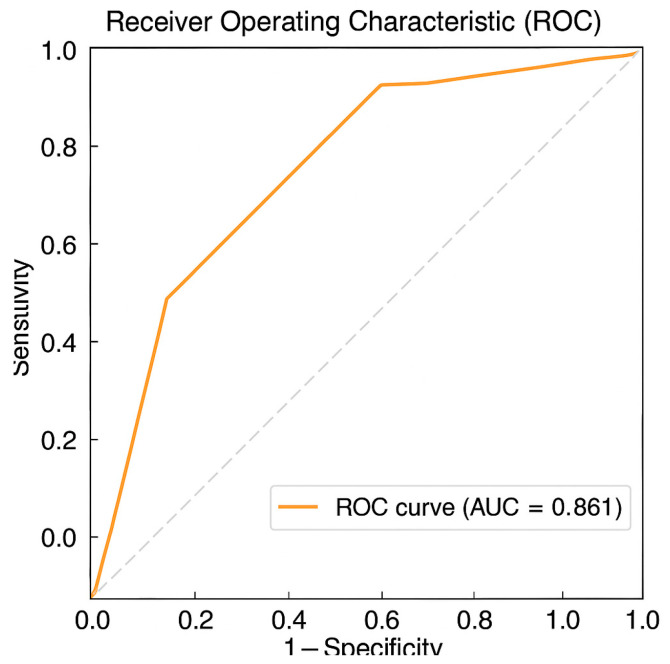
ROC curve for the GLMM showing sensitivity vs. 1-specificity for predicting ≥50% seizure reduction. AUC = 0.861.

The model demonstrated good overall fit with a −2 log-likelihood of 92.3, AIC of 100.3, and BIC of 109.1. Inclusion of stimulation parameters significantly improved fit over baseline models lacking these variables. Receiver operating characteristic (ROC) analysis yielded an area under the curve (AUC) of 0.861 (95% CI: 0.768–0.953), indicating excellent model discrimination. Calibration was acceptable (Hosmer–Lemeshow *p* = 0.38) (Figure 3).

A supplementary heatmap figure (Supplementary Figure S1) was added to depict the combined relationship of output current, duty cycle, and clinical response. This visualization highlights the optimal range of ~1.5 mA and 10% duty cycle.

### 3.6. Visualization of Stimulation-Response Relationship

To further illustrate the relationship between stimulation parameters and clinical response, a supplementary analysis was conducted. A scatter plot of responders and non-responders by final output current and duty cycle demonstrated clustering of responders around ~1.5 mA and 10% duty cycle. To enhance interpretability, this visualization has been reformatted as a heatmap (Supplementary Figure S1), which more clearly depicts the combined distribution of clinical response across parameter ranges.

The supplementary figure highlights a concentration of responders within the 1.25–1.75 mA and 10–15% duty cycle range, supporting the presence of an optimal stimulation window. Beyond these thresholds, the likelihood of additional benefit appeared to plateau.

## 4. Discussion

This two-center retrospective study investigated the efficacy of VNS in pediatric patients with DRE, with a specific focus on identifying optimal stimulation parameters. The findings demonstrate that VNS is associated with significant clinical benefit in this population, with 76.9% of patients achieving a ≥50% reduction in seizure frequency at 12-month follow-up. Moreover, 32.7% attained ≥90% seizure reduction, and 11.5% achieved complete seizure freedom. Importantly, the study was retrospective in nature, and results should be interpreted as associations rather than causal effects. Notably, stimulation parameters—particularly output current and duty cycle—were found to be independent predictors of clinical response, with optimal outcomes clustered around an output current of 1.5 mA and a duty cycle of 10%.

These results align with and expand upon prior findings in the literature. For example, the CORE-VNS study, which included a large cohort of pediatric patients with Lennox–Gastaut syndrome, reported a 47.4% responder rate at 12 months, despite utilizing higher stimulation intensities on average [18]. Similarly, a meta-analysis by Wei et al. evaluating VNS in children with Dravet syndrome found highly variable seizure outcomes across studies, with no consistent dose–response relationship identified [19]. In contrast, our study suggests that clinically meaningful seizure control may be achieved in pediatric patients using more conservative stimulation protocols, thereby reducing the risk of overstimulation-related adverse effects such as hoarseness, dysphagia, and sleep disruption [20].

The GLMM analysis demonstrated a linear relationship between stimulation intensity and clinical response, whereas visual inspection of scatter and heatmap plots suggested a plateau effect at ~1.5 mA and 10% duty cycle. This apparent discrepancy likely reflects sample distribution and ceiling effects and has been acknowledged as a methodological limitation.

The significance of stimulation parameters as independent predictors of response is consistent with previous studies in both pediatric and adult populations [21,22]. However, the clustering of responders within a relatively narrow stimulation window highlights the importance of avoiding unnecessary escalation, which may not yield further benefit and may increase side-effect risk. These findings are congruent with earlier reports suggesting that younger brains, due to greater neuroplasticity and lower activation thresholds, may respond to lower-intensity stimulation [23,24].

From a mechanistic standpoint, the enhanced efficacy at lower stimulation intensities in pediatric patients may reflect differences in vagal afferent density, synaptic integration, or central autonomic responsiveness during neurodevelopment [25]. Functional neuroimaging studies have indicated broader and more diffuse cortical activation in children undergoing VNS compared to adults, which may partly explain this heightened sensitivity to stimulation [26]. Future studies incorporating electrophysiological monitoring (e.g., EEG, heart rate variability) or functional neuroimaging are warranted to provide mechanistic validation of these clinical findings.

Clinically, the results support a shift away from adult-derived titration strategies toward pediatric-specific programming protocols. Early stabilization at an output current of ~1.5 mA and a duty cycle of 10% may serve as a practical and well-tolerated programming target, avoiding unnecessary increases that may not yield additional benefit. This is particularly important in syndromic epilepsies or in patients with comorbidities who may be more susceptible to stimulation-related side effects.

The strengths of this study include the standardized stimulation parameters (frequency and pulse width), a relatively homogenous cohort with robust follow-up, and the use of GLMM to account for inter-individual variability. Moreover, the identification of a clear stimulation-response window has direct translational relevance for clinical practice.

Several limitations should be acknowledged. First, the modest sample size, while sufficient for main analyses, precluded subgroup comparisons by epilepsy syndrome, etiology, or age strata. Second, the retrospective study design and reliance on caregiver-maintained seizure diaries introduce potential reporting and recall bias, and prospective validation is needed. Third, adverse events were not systematically recorded; thus, safety and tolerability conclusions should be interpreted cautiously, although clinical notes occasionally documented transient hoarseness and mild dysphagia. Fourth, the study was conducted at two tertiary centers in Türkiye, which may limit the generalizability of the findings to other healthcare systems and populations. Finally, although GLMM modeling identified stimulation parameters as predictors of response, the apparent plateau effect in visual analyses indicates that the true dose–response relationship may be more complex than a linear model suggests.

In conclusion, this study highlights the clinical utility of individualized, age-appropriate stimulation protocols in pediatric VNS therapy. A parameter combination of approximately 1.5 mA output current and 10% duty cycle appears sufficient to elicit meaningful seizure reduction in the majority of pediatric patients. These results support a paradigm shift toward more conservative and evidence-informed titration strategies that prioritize both efficacy and tolerability.

## 5. Conclusions

This two-center retrospective analysis reinforces the clinical effectiveness and tolerability of VNS in children with drug-resistant epilepsy. The findings demonstrate that a stimulation regimen centered on an output current of approximately 1.5 mA and a duty cycle of 10% is sufficient to elicit significant seizure reduction in the majority of pediatric patients. Early attainment and stabilization at this threshold were associated with improved outcomes, suggesting these parameters may serve as practical targets in pediatric VNS programming.

Importantly, the study supports a shift toward individualized titration strategies tailored to the unique physiological and neurodevelopmental characteristics of children, rather than defaulting to adult-based stimulation protocols. Conservative, parameter-specific approaches may enhance therapeutic efficacy while reducing the risk of stimulation-related side effects. Future prospective, multicenter studies with larger and more diverse pediatric cohorts are warranted to confirm these findings and guide the development of standardized, age-appropriate VNS treatment algorithms.

## Figures and Tables

**Table 1:** Baseline demographic and clinical characteristics of study cohort.

Variable	Value (Mean ± SD or n (%))
Number of patients	52
Age at implantation (years)	9.2 ± 4.5
Epilepsy duration (years)	5.8 ± 3.1
Male sex	30 (57.7%)
Female sex	22 (42.3%)
Etiology	Genetic: 17 (32.7%), Structural: 11 (21.2%), Infectious: 3 (5.8%), Other: 21 (40.3%)
Seizure type	Focal: 24 (46.1%), Generalized: 28 (53.9%)
Baseline seizure frequency (per month)	54.7 ± 21.3
Initial output current (mA)	0.25
Initial duty cycle (%)	10
Stimulation frequency (Hz)	30
Pulse width (μs)	250

Data are presented as mean ± standard deviation (SD) or n (%). Normality of continuous variables was assessed.

**Table 2:** Initial and maximum VNS parameters.

Parameter	Initial Value	Final Value (12 Months)
Output current (mA)	0.25	1.5 (range 0.75–2.25)
Duty cycle (%)	10	20 (range 10–30)
Frequency (Hz)	30	30
Pulse width (μs)	250	250

Stimulation parameters (output current and duty cycle) were titrated individually based on clinical response, while frequency and pulse width were held constant.

**Table 3:** Clinical outcomes at 12 months post-implantation.

Outcome Category	Patients (n)	Proportion (%)
≥50% seizure reduction	40	76.9%
≥90% seizure reduction	17	32.7%
Seizure freedom	6	11.5%

Clinical outcomes are reported as frequencies and percentages. Response was defined as ≥50% reduction in seizure frequency relative to baseline. No inferential comparisons were made; results are intended to reflect observed outcomes descriptively.

**Table 4:** GLMM results for predictors of clinical response.

Predictor	OR	95% CI	*p*-Value
Output current (mA)	4.48	2.29–8.76	**<0.001**
Duty cycle (%)	1.11	1.04–1.27	**0.002**
Age at implantation (yrs)	1.02	0.95–1.10	0.571
Epilepsy duration (yrs)	0.95	0.84–1.08	0.446

A generalized linear mixed model (GLMM) with a logit link function was employed to evaluate predictors of clinical response (defined as a ≥50% reduction in seizure frequency). Fixed effects included output current, duty cycle, age at implantation, and epilepsy duration. Random intercepts accounted for subject-level variability. Odds ratios (ORs) with 95% confidence intervals (CIs) are presented. Statistical significance was defined as *p* < 0.05. Model fit was assessed using −2 log-likelihood, Akaike Information Criterion (AIC), and Bayesian Information Criterion (BIC).

## Data Availability

The datasets generated and analyzed during the current study are not publicly available due to institutional data protection regulations but are available from the corresponding author upon reasonable request.

## Supplementary Materials

The following supporting information can be downloaded at: https://www.mdpi.com/article/10.3390/children12091222/s1, Figure S1: Heatmap of response by stimulation parameters.

## Abbreviations

The following abbreviations are used in this manuscript:

DRE	Drug-resistant epilepsy
VNS	Vagus nerve stimulation
ASMs	Antiseizure medications
ILAE	International League Against Epilepsy
SUDEP	Sudden unexpected death in epilepsy
µs	Microseconds
GLMM	Generalized linear mixed model
AIC	Akaike Information Criterion
BIC	Bayesian Information Criterion
OR	Odds ratio
CI	Confidence intervals
ROC	Receiver operating characteristic
AUC	Area under the curve
