# Supplementary figures and images for: Optimizing Vagus Nerve Stimulation Parameters in Pediatric Drug-Resistant Epilepsy: A Retrospective Two-Center Study
Source: Children (Basel). 2025 Sep 12;12(9):1222. doi: 10.3390/children12091222 (PMC12468347; doi:10.3390/children12091222)

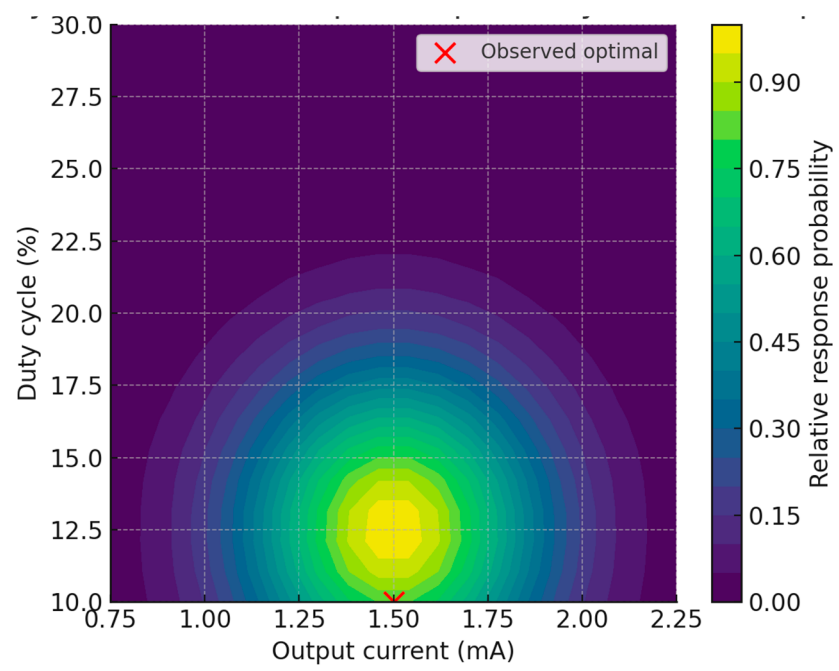

**Supplementary Figure S1.** Heatmap of response by stimulation parameters

Supplement: Supplementary file 1 [file children-12-01222-s001.zip › children-3814402-supplementary.pdf]
